# First Total Synthesis and Pharmacological Potential of a Plant Based Hexacyclopeptide 

**DOI:** 10.22037/ijpr.2019.1100643

**Published:** 2019

**Authors:** Rajiv Dahiya, Sunil Singh, Sheeba Varghese Gupta, Vijaykumar B. Sutariya, Deepak Bhatia, Rita Mourya, Suresh V. Chennupati, Ajay Sharma

**Affiliations:** a *Laboratory of Peptide Research and Development,* *School of Pharmacy, Faculty of Medical Sciences, The University of the West Indies, St. Augustine, Trinidad & Tobago, West Indies.*; b *Department of Pharmacy, Faculty of Pharmaceutical Science, Mewar University, Gangrar, Chittorgarh, Rajasthan, India.*; c *Department of Pharmaceutical Sciences, USF College of Pharmacy, University of South Florida, Tampa, FL 33612-4749, USA.*; d *Department of Pharmacogenomics, Bernard J. Dunn School of Pharmacy, Shenandoah University- ICPH Fairfax, Fairfax, VA 22031, USA.*; e *School of Pharmacy, College of Medicine and Health Sciences, University of Gondar, Gondar 196, Ethiopia. *; f *Department of Pharmacy, College of Medical and Health Sciences, Wollega University, P.O. Box 395, Nekemte, Ethiopia. *; g *Department of Pharmacognosy, Amity Institute of Pharmacy, Amity University, Gwalior, Madhya Pradesh, India.*

**Keywords:** Diandrine C, Proline-rich cyclic peptide, Solution-phase peptide synthesis, Drymaria diandra, Antimicrobial activity, Antihelmintic activity

## Abstract

A new bioactive proline-rich cyclohexapeptide - diandrine C (**6**), previously isolated from whole plant of *Drymaria diandra* (Caryophyllaceae), was synthesized through coupling reactions of tetrapeptide unit Boc-Gly--Pro--Tyr--Trp-OH with dipeptide unit -Pro-Gly-OMe using *N,N*-diisopropylcarbodiimide (DIPC) as the coupling agent, followed by cyclization of linear hexapeptide unit under alkaline condition. Structure of cyclohexapeptide was confirmed by means of chemical, and spectroscopic analyses and also was screened for its antimicrobial and anthelmintic properties. Bioevaluation results indicated that the newly synthesized hexacyclopeptide exhibited potent antimicrobial activity against Gram-negative bacteria *Pseudomonas aeruginosa*, *Klebsiella pneumoniae* and pathogenic *Candida albicans *at 6 μg/mL. Moderate to good level of antihelmintic activity against three earthworm species *Megascoplex konkanensis*, *Pontoscotex corethruses* and *Eudrilus eugeniae* was also observed at concentration of 2 mg/mL.

## Introduction

Plants are vital source of therapeutic agents with diverse biological properties (1, 2). Over 80% of the global population rely on traditional medicine, much of which is based on plant remedies. Natural products from medicinal plants, either as pure compounds or as standardized extracts, provide unlimited opportunities for new drug leads because of the unmatched availability of chemical diversity (3). Since decades, drug development based on plant-derived natural products has been remained interesting and challenging (4) and plant based-cyclopolypeptides played a vital role in drug design and has provided significant promise for future endeavours (5). They have complex structures with modified amino acid moieties and are associated with a number of pharmacological activities including antifungal, tyrosinase inhibitory, anti-inflammatory, antimalarial, protease inhibitory, anthelmintic, and antineoplastic activity (6-12). Diandrines are glycine and proline-rich cyclopolypeptides consisting of 6-8 amino acid units, existing as stable conformational isomers. Diandrines are isolated from the methanolic extract of Formosan *Drymaria diandra *(13). These compounds are known for their selective inhibitory effect on collagen-induced platelet aggregation*.* Researchers were unable to investigate the biological properties of cyclopeptides in detail due to availability of only minute quantities of cyclopeptides from natural resources. 

Keeping in view the array of bioactivities possessed by proline-rich peptides and other cyclooligopeptides (5, 14) and further, in continuation of efforts of our research unit (15-17) for synthesizing cyclopolypeptides of biological interest in quantitative yield, this study was directed toward an effective solution-phase synthesis with good yield, along with structure elucidation and screening of hexacyclopeptide - diandrine C for antibacterial, antifungal, and anthelmintic potential.

## Experimental

*Chemistry*

-Amino acids, di-*tert*-butylpyrocarbonate (Boc), pentafluorophenol (*pfp*), *N,N*-diisopropylcarbodiimide (DIPC) and *N*-methylmorpholine (NMM) were obtained from Spectrochem Limited, Mumbai, India. IR spectra were recorded on Shimadzu 8700 Fourier transform infrared spectrophotometer using a thin film supported on KBr pellets for hexacyclopeptide and CHCl_3_ as solvent for intermediate semisolids. ^1^H-NMR and ^13^C-NMR spectra were recorded on Bruker AC NMR spectrometer (300 MHz) using CDCl_3 _as solvent and tetramethylsilane (TMS) as internal standard. Mass spectra were recorded on Jeol JMS DX 303 Mass spectrometer operating at 70 eV. Elemental analysis of all compounds was performed on Elementar vario EL III. Purity of all the compounds was checked by TLC on precoated silica gel G plates. 

*General method for the synthesis of dipeptide units (* ***1-3*** *)*

Amino acid methyl ester hydrochloride (0.01 mol) was dissolved in chloroform (20 mL). Triethylamine (TEA, 2.8 mL, 0.021 mol) was added to above solution at 0 °C and resulting reaction mixture was stirred for 15 min. To this, another mixture of Boc-amino acid (0.01 mol) in chloroform (20 mL) and DIPC (1.26 g, 0.01 mol) was added with stirring. After 24 h, the final reaction mixture was filtered and the filtrate was washed with 5% NaHCO_3 _and saturated NaCl solutions. The organic layer was dried over anhydrous Na_2_SO_4_, filtered, and evaporated in vacuum. The crude product was recrystallized from a mixture of chloroform and petroleum ether.

*tert-butyloxycarbonyl-glycyl-l-proline methyl ester* *(****1****)*

Semi-solid mass, yield 79.4%, R_f_ - 0.79 (CHCl_3_:MeOH (7:3, *v/v*).

IR (CHCl_3_):* v *3125 (*m*, -NH str, amide), 2999, 2994 (*m*, -CH str, cyclic CH_2_ and CH), 2929 (*m*, -CH str, asym, CH_2_), 2845 (*m*, -CH str, sym, CH_2_), 1754 (*s*, -C=O str, ester), 1672, 1635 (*s*, -C=O str, 3° and 2° amide), 1535 (*m*, -NH bend, 2° amide), 1392, 1360 (*m*, -CH bend, *t*-butyl group), 1272 (*s*, C−O str, ester) cm^-1^.

^1^H NMR (CDCl_3_, 300 MHz): δ 6.39 (1H, *br. s*, -NH), 4.29-4.26 (1H, *t*, α -H, Pro), 3.75-3.72 (2H, *t*, δ-H’s, Pro), 3.62 (3H, *s*, OCH_3_), 3.50-3.48 (2H, *d*, *J* = 4.8 Hz, CH_2_, Gly), 2.08-2.02 (2H, *m*, β-H’s, Pro), 1.99-1.93 (2H, *m*, γ-H’s, Pro), 1.55 (9H, *s*, *t*-butyl group) ppm.

Found: C, 54.52; H, 7.77; N, 9.76; C_13_H_22_N_2_O_5_ requires C, 54.53; H, 7.74; N, 9.78%.

*tert-butyloxycarbonyl-l-tyrosinyl-l-tryptophan methyl ester* *(****2****)*

Semisolid mass, yield 71.5%, R_f_ - 0.87 (CHCl_3_: MeOH (7:3, *v/v*).

IR (CHCl_3_):* v *3473 (*m*, -NH str, indole ring), 3379 (*m*, -OH str, Tyr), 3077-3069 (*w*, -CH str, aromatic rings), 2852, 2848 (*m*, -CH str, sym, CH_2_), 1751 (*s*, -C=O str, ester), 1639, 1635 (*s*, -C=O str, 2° amide), 1558, 1552, 1423-1419 (*m*, skeletal bands, aromatic rings), 1535-1531 (*m*, -NH bend, 2° amide), 1390, 1365 (*m*, -CH bend, *t*-butyl group), 1269 (*s*, C−O str, ester), 714-709, 698, 692 (*s*, -CH bend, oop, aromatic rings) cm^-1^.

^1^H NMR (300 MHz, CDCl_3_): δ7.53-7.51 (1H, *d*, *J* = 7.75 Hz, -H, indole ring), 7.45 (2H, *br. s*, -NH, indole ring and -OH, Tyr), 7.33-7.29 (2H, *dd*, *J* = 8.6, 4.9 Hz, *m*-H’s, Tyr), 7.16-7.09 (3H, *m*, δ-ζ-H’s, indole ring), 7.05-7.03 (1H, *d*, *J* = 7.3 Hz, γ-H, indole ring), 6.93-6.89 (2H, *dd*, *J* = 8.6, 5.3 Hz, *o*-H’s, Tyr), 6.68 (1H, *br. s*, -NH, Trp), 6.65 (1H, *br. s*, -NH, Tyr), 4.89-4.84 (1H, *q*, *J* = 6.1 Hz, α -H, Trp), 4.72-4.68 (1H, *q*, *J* = 7.9 Hz, α -H, Tyr), 3.57 (3H, *s*, OCH_3_), 3.25-3.23 (2H, *d*, *J* = 5.5 Hz, β-H’s, Tyr), 3.13-3.11 (2H, *d*, *J* = 5.7 Hz, β-H’s, Trp), 1.55 (9H, *s*, *t*-butyl group) ppm.

Found: C, 64.84; H, 6.51; N, 8.75, C_26_H_31_N_3_O_6_ requires C, 64.85; H, 6.49; N, 8.73%.

*tert-butyloxycarbonyl-l-prolyl-l-glycine methyl ester (* ***3*** *)*

Semi-solid mass, yield 73.8%, R_f_ - 0.66 (CHCl_3_:MeOH (7:3, *v/v*).

IR (CHCl_3_): *v *3128 (*m*, -NH str, amide), 2998-2992 (*m*, -CH str, cyclic CH_2_ and CH), 2927(*m*, -CH str, asym, CH_2_), 2846 (*m*, -CH str, sym, CH_2_), 1752 (*s*, -C=O str, ester), 1673, 1638

(*s*, -C=O str, 3° and 2° amide), 1536 (*m*, -NH bend, 2° amide), 1390, 1363 (*m*, -CH bend, *t*-butyl group), 1275 (*s*, C−O str, ester) cm^-1^.

^1^H NMR (CDCl_3_, 300 MHz): δ6.37 (1H, *br. s*, -NH), 4.27-4.24 (1H, *t*, δ -H, Pro), 3.79-3.76

(2H, *t*, δ -H’s, Pro), 3.65 (3H, *s*, OCH_3_), 3.51-3.49 (2H, *d*, *J *= 4.7 Hz, CH_2_, Gly), 2.04-1.97 (4H, *m*, β-H’s and γ-H’s, Pro), 1.53 (9H, *s*, *t*-butyl group) ppm.

Found: C, 54.50; H, 7.75; N, 9.79; C_13_H_22_N_2_O_5 _requires C, 54.53; H, 7.74; N, 9.78%.

*General method for the synthesis of linear tetra/hexapeptide fragments (* ***4, 5*** *)*

A solution of Boc-di/tetrapeptide (0.01 mol) dissolved in 25 mL of *N,N*-dimethylformamide (DMF) was neutralized with 2.21 mL (0.021 mol) of *N*-methylmorpholine (NMM) at 0 °C, followed by stirring of the resulting mixture for 15 min. Dipeptide methyl ester (0.01 mol) was dissolved in 25 mL of DMF and resulting solution with DIPC (1.26 g, 0.01 mol) were added to the above mixture. Stirring was first done for 1 h at 0-5 °C and then, further for 24 h at RT. 

The reaction mixture was diluted with an equal amount of water and the semisolid mass obtained was washed with water and purified from a mixture of chloroform and petroleum ether (b.p. 40-60 °C) followed by cooling at 0 °C.

*tert-butyloxycarbonyl-glycyl-l-prolyl-l-tyrosinyl-l-tryptophan methyl ester (* ***4*** *)*

Semisolid mass, Yield 77.2%, R_f_ - 0.49 (CHCl_3_:MeOH (7:3, *v/v*).

IR (CHCl_3_): *v *3475 (*m*, -NH str, indole ring), 3377 (*m*, -OH str, Tyr), 3128-3123 (*m*, -NH str, amide), 3079-3071 (*w*, -CH str, aromatic rings), 2999-2992 (*m*, -CH str, cyclic CH2 and CH), 2929, 2926 (*m*, -CH str, asym, CH_2_), 2850, 2845 (*m*, -CH str, sym, CH_2_), 1751 (*s*, -C=O str, ester), 1668, 1636, 1632 (*s*, -C=O str, 3° and 2° amide), 1559, 1553, 1425-1419 (*m*, skeletal bands, aromatic rings), 1539, 1534 (*m*, -NH bend, 2° amide), 1395, 1363 (*m*, -CH bend, *t*7 butyl group), 1269 (*s*, C−O str, ester), 718-712, 699, 692 (*s*, -CH bend, *oop*, aromatic rings) cm-1.

^1^H NMR (300 MHz, CDCl_3_): δ 7.52-7.50 (1H, *d*, *J *= 7.8 Hz, indole ring), 7.42 (2H, *br. s*, -NH, indole ring and -OH, Tyr), 7.18-7.09 (3H, *m*-H’s, indole ring), 7.04-7.02 (1H, *d*, *J *= 7.3 Hz, γ -H, indole ring), 6.99-6.95 (2H, *dd*, *J *= 8.6, 4.9 Hz, *m*-H’s, Tyr), 6.93 (1H, *br. s*, - NH, Trp), 6.89-6.85 (2H, *dd*, *J *= 8.7, 5.3 Hz, *o*-H’s, Tyr), 6.54 (1H, *br. s*, -NH, Tyr), 6.38 (1H, *br. s*, -NH, Gly), 5.01-4.97 (1H, *q*, *J *= 7.9 Hz, -H, Tyr), 4.46-4.43 (1H, *t*, -H, Pro), 4.23-4.19 (1H, *q*, *J *= 6.2 Hz, -H, Trp), 3.69-3.66 (2H, *t*, δ-H’s, Pro), 3.54 (3H, *s*, OCH_3_), 3.51-3.49 (2H, *d*, *J *= 4.8 Hz, CH_2_, Gly), 3.26-3.24 (2H, *d*, *J *= 5.7 Hz, β-H’s, Trp), 2.93-2.91 (2H, *d*, *J *= 5.6 Hz, β-H’s, Tyr), 2.69-2.63 (2H, *m*, β-H’s, Pro), 1.96-1.92 (2H, *m*, γ-H’s, Pro), 1.51 (9H, *s*, *t*-butyl group) ppm.

Found: C, 62.33; H, 6.49; N, 10.99; C_33_H_41_N_5_O_8_ requires C, 62.35; H, 6.50; N, 11.02%.

*tert-butyloxycarbonyl-glycyl-l-prolyl-l-tyrosinyl-l-tryptophanyl-l-prolyl-glycine methyl ester(* ***5*** *)*

Semisolid mass, Yield 81.7%, R_f_ - 0.71 (CHCl_3_:MeOH (9:1, *v/v*).

IR (CHCl_3_): *v *3472 (*m*, -NH str, indole ring), 3375 (*m*, -OH str, Tyr), 3129-3122 (*m*, -NH str, amide), 3077-3072 (*w*, -CH str, aromatic rings), 2999, 2997-2991 (*m*, -CH str, cyclic CH_2_ and CH), 2929, 2927-2924 (*m*, -CH str, asym, CH_2_), 2849, 2846-2842 (*m*, -CH str, sym, CH_2_), 1753 (*s*, -C=O str, ester), 1672-1668, 1636-1632 (*s*, -C=O str, 3° and 2° amide), 1557-1553, 1427-1422 (*m*, skeletal bands, aromatic rings), 1537, 1533 (*m*, -NH bend, 2° amide), 1391, 1366 (*m*, -CH bend, *t*-butyl group), 1272 (*s*, C−O str, ester), 719-715, 697, 690 (*s*, -CH bend, oop, aromatic rings) cm^-1^.

^1^H NMR (300 MHz, CDCl_3_): δ 8.63 (1H, *br. s*, -NH, Gly-2), 7.96 (1H, *br. s*, -NH, Trp), 7.46 (2H, *br. s*, -NH, indole ring and -OH, Tyr), 7.39-7.37 (1H, *d*, *J *= 7.8 Hz, α -H, indole ring), 7.21-7.19 (1H, *d*, *J *= 7.3 Hz, γ -H, indole ring), 7.15-7.08 (3H, *m*, δ -H’s, indole ring), 6.97- 6.93 (2H, *dd*, *J *= 8.7, 4.8 Hz, *m*-H’s, Tyr), 6.90-6.86 (2H, *dd*, *J *= 8.7, 5.3 Hz, *o*-H’s, Tyr), 6.55 (1H, *br. s*, -NH, Tyr), 6.36 (1H, *br. s*, -NH, Gly-1), 4.83-4.79 (1H, *q*, *J *= 7.8 Hz, α -H, Tyr), 4.48-4.43 (2H, *m*, α -H, Trp and α -H, Pro-1), 4.08-4.05 (1H, *t*, δ -H, Pro-2), 4.03-4.01 (2H, *d*, *J *= 4.8 Hz, CH_2_, Gly-2), 3.71-3.68 (2H, *t*, δ-H’s, Pro-1), 3.62 (3H, *s*, OCH_3_), 3.55- 3.53 (2H, *d*, *J *= 4.8 Hz, CH_2_, Gly-1), 3.34-3.31 (2H, *t*, δ-H’s, Pro-2), 3.21-3.19 (2H, *d*, *J *= 5.7 Hz, β-H’s, Trp), 2.95-2.93 (2H, *d*, *J *= 5.6 Hz, β-H’s, Tyr), 2.69-2.62 (4H, *m*, β-H’s, Pro-1 and Pro-2), 1.98-1.91 (4H, *m*, γ-H’s, Pro-1 and Pro-2), 1.53 (9H, *s*, *t*-butyl group) ppm. ^13^C NMR (CDCl_3_, 300 MHz): δ 174.7 (C=O, Tyr), 170.2 (C=O, Pro-1), 169.4, 168.7 (2C, C=O, Gly-2 and Gly-1), 162.8 (C=O, Pro-2), 161.4 (C=O, Trp), 155.0 (C=O, Boc) 153.8 (*p*-C, Tyr), 135.5 (-C, indole ring), 133.0 (2C, *m*-C’s, Tyr), 130.9 (2C, *o*-C’s, Tyr), 129.6 (γ -C, Tyr), 128.2 (-C, indole ring), 123.9, 122.0 (2C, -C and, indole ring), 119.8, 118.3 (2C, δ-C and γ -C, indole ring), 112.4, 109.2 (2C, β, indole ring), 79.3 (-C, Boc), 69.1 (-C, Pro-2), 58.2 (-C, Trp), 55.2 (-C, Pro-1), 52.5 (-C, Tyr), 50.9 (OCH_3_), 47.1 (CH_2_, Gly-1), 45.4, 44.0 (2C, δ -C’s, Pro-1 and Pro-2), 39.4 (CH_2_, Gly-2), 37.2 (β-C, Tyr), 29.7 (β- C, Pro-1), 28.9 (3C, β-C’s, Boc), 27.5 (β-C, Pro-2), 25.5, 24.1 (2C, γ -C’s, Pro-2 and Pro-1), 22.9 (β-C, Trp) ppm.

Found: C, 60.85; H, 6.53; N, 12.39; C_40_H_51_N_7_O_10_ requires C, 60.82; H, 6.51; N, 12.41%.

*Procedure for the synthesis of cyclic hexapeptide, diandrine C (* ***6*** *)*

In order to carry out the synthesis of cyclopeptide (**6**), linear hexapeptide unit (**5, **0.005 mol) was deprotected at carboxyl end using LiOH (0.18 g, 0.0075 mol) to get Boc-Gly*-l-*Pro*-l*-Tyr*-l-*Trp*-l-*Pro-Gly-OH.The deprotected hexapeptide unit (0.005 mol) was now dissolved in CHCl_3_ (50 mL) at 0 °C. To the above solution, pentafluorophenol (1.23 g, 0.0067 mol) and DIPC (0.63 g, 0.005 mol) was added and stirred at RT for 12 h. The reaction mixture was filtered and the filtrate was washed with 10% NaHCO_3_ solution (2 × 25 mL) and 5% HCl (3 × 15 mL) to get the corresponding pentafluorophenyl ester Boc-Gly*-l-*Pro*-l-*Tyr*-l-*Trp*-l-*Pro-Gly-O-*pfp*. To this compound (0.004 mol) dissolved in chloroform (25 mL), trifluoroacetic acid (TFA, 0.91 g, 0.008 mol) was added, stirred at RT for 1 h, and washed with 10% NaHCO_3_ solution (3 × 20 mL). The organic layer was dried over anhydrous Na_2_SO_4 _to get Gly*-l-*Pro*-l-*Tyr*-l-*Trp*-l-*Pro-Gly-O-*pfp, *which was dissolved in CHCl_3_ (25 mL) and also, TEA (2.8 mL, 0.02 mol) was added. Then, whole content was kept for 1 week time at 0 °C. This step of cyclization was repeated separately by addition of NMM (2.21 mL, 0.02 mol) and then, by addition of pyridine (1.61 mL, 0.02 mol) to Gly*-l-*Pro*-l-*Tyr*-l-*Trp*-l-*Pro-Gly-O-*pfp*. In all the three cases of cyclization, the reaction mixture was washed with 10% NaHCO_3_ and 5% HCl solutions (3 × 25 mL) individually. 

The organic layer was dried over anhydrous Na_2_SO_4_. Finally, chloroform was distilled off and crude cyclized product was crystallized from CHCl_3_/*n*-hexane to get pure cyclo (*glycyl-l-prolyl-l-tyrosinyl-l-tryptophanyl-l-prolyl-glycyl)*(**6**).

Pale yellow needles, m.p. 114-115 °C, yield: 2.74 g, 83.2% (NMM), 2.49 g, 75.7% (TEA), 2.27 g, 68.9% (C_5_H_5_N), [α]_D_: +2.1° (+2.2°) (MeOH, c 0.19), R_f_ - 0.84 (CHCl_3_:MeOH (9:1, *v/v*).

IR (KBr): *v *3476 (*m*, -NH str, indole ring), 3372 (*m*, -OH str, Tyr), 3127, 3125-3122 (*m*, -NH str, amide), 3075, 3072 (*w*, -CH str, aromatic rings), 2997, 2994-2989 (*m*, -CH str, cyclic CH_2_ and CH), 2928, 2925-2922 (*m*, -CH str, asym, CH_2_), 2848-2845, 2842 (*m*, -CH str, sym, CH_2_), 1674, 1669, 1635-1632 (*s*, -C=O str, 3° and 2° amide), 1555-1552, 1425-1421 (*m*, skeletal bands, aromatic rings), 1539, 1535 (*m*, -NH bend, 2 ° amide), 721-717, 695- 689 (*s*, -CH bend, *oop*, aromatic rings) cm^-1^.

^1^H NMR (300 MHz, CDCl_3_): δ 9.85 (1H, *br. s*, -NH, Tyr), 9.16 (1H, *br. s*, -NH, Gly-2), 7.65 (1H, *br. s*, -NH, Trp), 7.42 (2H, *br. s*, -NH, indole ring and -OH, Tyr), 7.41-7.39 (1H, *d*, *J *= 7.8 Hz, α -H, indole ring), 7.25-7.23 (1H, *d*, *J *= 7.3 Hz, γ-H, indole ring), 7.16-7.07 (3H, *m*, δ -H’s, indole ring), 6.99-6.95 (2H, *dd*, *J *= 8.6, 4.8 Hz, *m*-H’s, Tyr), 6.92-6.88 (2H, *dd*, *J *= 8.7, 5.3 Hz, *o*-H’s, Tyr), 6.26 (1H, *br. s*, -NH, Gly-1), 5.78-5.74 (1H, *q*, *J *= 6.2 Hz, α -H, Trp),5.31-5.29 (2H, *d*, *J *= 4.7 Hz, CH_2_, Gly-2), 4.23-4.19 (1H, *q*, *J *= 7.8 Hz, α -H, Tyr), 3.96-3.94 (2H, *d*, *J *= 4.8 Hz, CH_2_, Gly-1), 3.91-3.86 (2H, *m*, α -H’s, Pro-1 and Pro-2), 3.27-3.21 (4H, *m*, δ -H’s, Pro-1 and Pro-2), 2.90-2.88 (2H, *d*, *J *= 5.7 Hz, β-H’s, Trp), 2.69-2.63 (4H, *m*, β-H’s,Pro-1 and Pro-2), 2.61-2.59 (2H, *d*, *J *= 5.7 Hz, β-H’s, Tyr), 1.88-1.79 (4H, *m*, γ-H’s, Pro-1 and Pro-2) ppm. ^13^C NMR (CDCl_3_, 300 MHz): δ 173.2, 172.9 (2C, C=O, Tyr and Pro-2), 171.2, 169.9 (2C, C=O, Pro-1 and Trp), 164.8, 163.2 (2C, C=O, Gly-2 and Gly-1), 154.0 (*p*-C, Tyr), 136.7 (α-C, indole ring), 133.9 (γ -C, Tyr), 130.2 (2C, *o*-C’s, Tyr), 128.7 (2C, *m*-C’s, Tyr), 126.7 (-C, indole ring), 125.5, 125.9 (2C, α -C and , indole ring), 120.4, 118.9 (2C, δ -C and γ -C, indole ring), 111.8, 110.3 (2C, β-C and -C, indole ring), 65.4 (α-C, Pro-2), 58.0 (α -C, Pro-1), 57.5 (α -C, Trp), 52.8 (α -C, Tyr), 49.7 (CH_2_, Gly-1), 49.1, 47.0 (2C, δ -C’s, Pro-2 and Pro-1), 42.4 (CH_2_, Gly-2), 37.7 (β-C, Tyr), 33.3 (β-C, Pro-1), 31.5 (β-C, Pro-2), 26.7 (β-C, Trp), 25.0, 23.3 (2C, γ -C’s, Pro-2 and Pro-1) ppm.

FAB MS: *m/z *658.7 (M + H)^+^, 630.7 (658.7–CO)^+^, 601.6 (Gly-Pro-Tyr-Trp-Pro)^+^, 573.6 (601.6–CO)^+^, 561.6 (Tyr-Trp-Pro-Gly-Gly)^+^, 533.6 (561.6–CO)^+^, 504.5 (Tyr-Trp-Pro-Gly)^+^, 476.5 (504.5–CO)^+^, 472.5 (Pro-Gly-Gly-Pro-Tyr)^+^, 447.5 (Tyr-Trp-Pro)^+^, 444.5 (472.5–CO)^+^, 419.5 (447.5–CO)^+^, 375.4 (Gly-Gly-Pro-Tyr)^+^, 350.4 (Tyr-Trp)^+^, 347.4 (375.4–CO)^+^, 322.4 (350.4–CO)^+^, 318.3 (Gly-Pro-Tyr)^+^, 309.3 (Pro-Gly-Gly-Pro)^+^, 290.3 (318.3–CO)^+^, 281.3 (309.3–CO)^+^, 212.2 (Pro-Gly-Gly)^+^, 184.2 (212.2–CO)^+^, 164.2 (Tyr)^+^, 159.2 (C_10_H_11_N_2_)^+^, 155.2 (Pro-Gly)^+^, 136.2 (C_8_H_10_NO)^+^, 130.1 (C_9_H_8_N)^+^, 127.2 (155.2–CO)^+^, 116.1 (C_8_H_6_N)^+^, 115.1 (Gly-Gly)^+^, 107.1 (C_7_H_7_O)^+^, 98.1 (Pro)^+^, 93.1 (C_6_H_5_O)^+^, 70.1 (C_4_H_8_N)^+^, 30.0 (CH_4_N)^+^.

Found: C, 62.08; H, 5.97; N, 14.89; C_34_H_39_N_7_O_7_ requires C, 62.09; H, 5.98; N, 14.91%.

*Biological activity studies*

Synthesized linear and cyclohexapeptide (**5, 6**) was screened for *in-vitro *antimicrobial activity against Gram-positive bacteria *Staphylococcus aureus (S. aureus), *Gram-negative bacteria *Pseudomonas aeruginosa (P. aeruginosa), Klebsiella pneumoniae *(*K. pneumoniae*) and *Escherichia coli (E. coli), *dermatophytes *Microsporum audouinii (M. audouinii)*, *Trichophyton mentagrophytes (T. mentagrophytes)*, diamorphic fungi *Candida albicans (C. albicans) *and *Aspergillus niger *(*A. niger*) at 50-6.25 μg/mL concentration using modified Kirby-Bauer disk diffusion method (18). MIC values of test compounds were determined by tube dilution technique. Gatifloxacin and griseofulvin/amphotericin B were used as reference drugs and DMF/DMSO were used as control. The results of antimicrobial activity studies are compiled in Table 1.

Compounds **5 **and **6 **were further screened for antihelmintic activity against earthworms *Eudrilus sp., Megascoplex konkanensis *and *Pontoscotex corethruses *at 2 mg/mL concentration using Garg’s method (19). Tween 80 (0.5%) in distilled water was used as control and mebendazole/piperazine citrate were used as standard drugs. The results of antihelmintic screening are tabulated in Table 2.

The detailed experimental procedures for pharmacological screening are already published in our previous reports (20-24).

## Results and Discussion

*Chemistry*

In order to carry out the synthesis of diandrine C (**6**), the cyclic hexapeptide molecule was split into three dipeptide units Boc-Gly-*l*-Pro-OMe (**1**), Boc-*l*-Tyr-*l*-Trp-OMe (**2**) and Boc-*l*-Pro-Gly-OMe (**3**). The required dipeptide units (**1-3**) were prepared by coupling of Boc-amino acids *viz. *Boc-Gly, Boc-*l*-Tyr and Boc-*l*-Pro with corresponding amino acid methyl ester hydrochlorides such as *l*-Pro-OMe·HCl, *l*-Trp-OMe·HCl and Gly-OMe·HCl employing diisopropylcarbodiimide (DIPC) as coupling agent following by the modified Bodanzsky and Bodanzsky method (25). Ester group of dipeptide (**1**) was cleaved by alkaline hydrolysis with LiOH and amino group of dipeptide (**2**) was deprotected by using trifluoroacetic acid (TFA).

**Table 1:** Antimicrobial activity data for linear and hexacyclopeptide (**5**, **6**).

**Compd.**	**Diameter of zone of inhibition (mm)**							
	# **Bacterial strains**				# **Fungal strains**			
	***S.*** ***aureus***	***K.*** ***pneumoniae***	***P.*** ***aeruginosa***	***E.*** ***coli***	***C.*** ***albicans***	***M.*** ***audouinii***	***A.*** ***niger***	***T.*** ***mentagrophytes***
**5** **6**	– 15 (25)^a^	22 (6) 26 (6)	19 (6) 22 (6)	11 (12.5) 13 (12.5)	23 (6) 27 (6)	10 (6) 14 (6)	– –	13 (6) 16 (6)
Control^b^	–	–	–	–	–	–	–	–
Gatifloxacin	27 (6)	25 (6)	23 (6)	20 (12.5)	–	–	–	–
Griseofulvin	–	–	–	–	–	18 (6)	–	20 (6)
Amphotericin B	–	–	–	–	25 (6)	–	21 (12.5)	–

a Values in brackets are MIC values (µg /mL);

b DMF/DMSO.

# *Staphylococcus aureus (S. aureus); Klebsiella pneumoniae (K. pneumoniae); Pseudomonas aeruginosa (P. aeruginosa); Escherichia coli (E. coli); Candida albicans (C. albicans); Microsporum audouinii (M. audouinii); Aspergillus niger (A. niger); Trichophyton mentagrophytes (T. mentagrophytes).*

**Table 2:** Antihelmintic activity data for linear and hexacyclopeptide (**5**, **6**)

**Compd.**	# **Earthworm species**					
	***M. konkanensis***		***P. corethruses***		***Eudrilus sp.***	
	**Mean paralyzing time (min)** a	**Mean death time (min)**	**Mean paralyzing time (min)**	**Mean death time (min)**	**Mean paralyzing time (min)**	**Mean death time (min)**
**5** b **6**^b^ Control^c^ Mebendazole^b^ Piperazine citrate^b^	13.57 ± 0.26 13.36 ± 0.37 – 10.52 ± 0.62 12.38 ± 0.49	15.18 ± 0.34 14.52 ± 0.14 – 12.57 ± 0.49 13.55 ± 0.27	21.56 ± 0.23 20.08 ± 0.42 – 18.02 ± 0.58 19.17 ± 0.44	24.06 ± 0.15 22.46 ± 0.33 – 19.49 ± 0.37 22.17 ± 0.26	16.09 ± 0.22 14.13 ± 0.27 – 11.29 ± 0.40 12.45 ± 0.19	17.57 ± 0.19 16.07 ± 0.46 – 13.37 ± 0.42 13.44 ± 0.36

a Data are given as mean ± SD (n = 3);

b c = 2 mg/mL;

c 0.5% Tween 80 in distilled water.

# *Megascoplex konkanensis (M. konkanensis); Pontoscotex corethruses (P. corethruses).*

**Figure 1:**
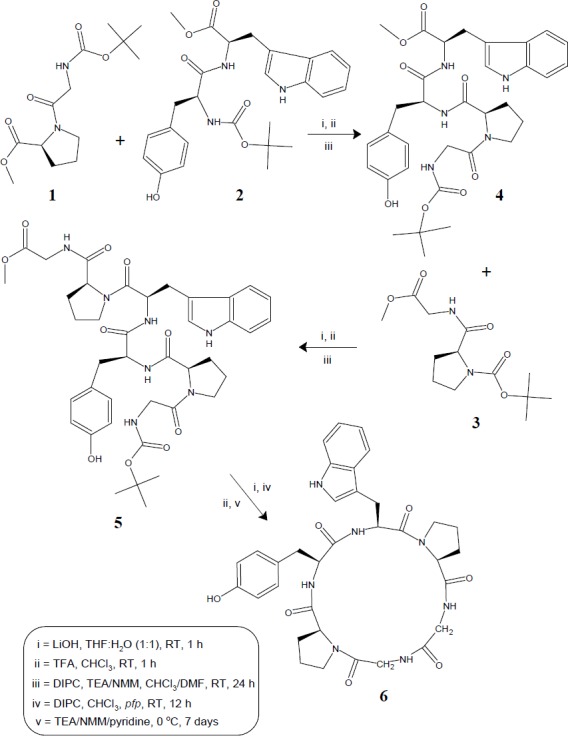
Synthetic route for hexacyclopeptide - diandrine C (**6**)

**Figure 2:**
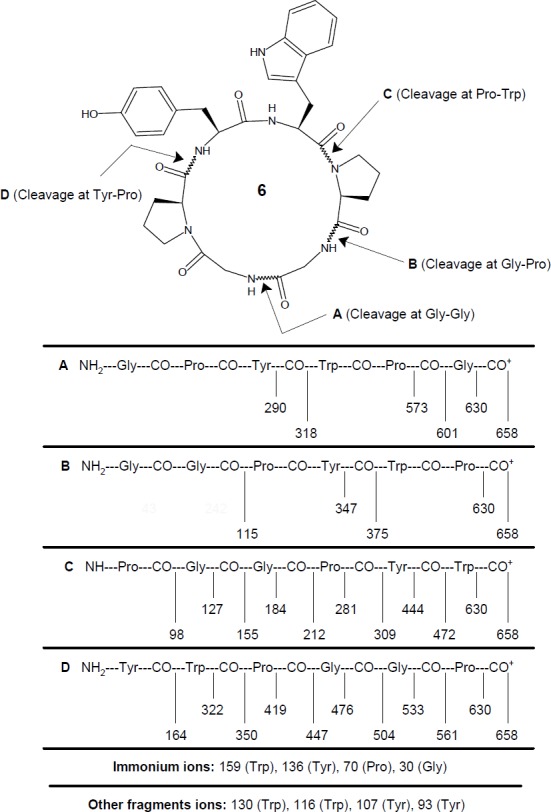
Mass fragmentation pattern for synthesized cyclohexapeptide diandrine C (**6**)

Both the deproptected dipeptides were coupled together using DIPC and *N*-methylmorpholine (NMM), to get the tetrapeptide unit Boc-Gly-*l*-Pro-*l*-Tyr-*l*-Trp-OMe (**4**). Similarly, dipeptide unit (**3**) after deprotection at amino end, was coupled with tetrapeptide (**4**) deprotected at carboxyl terminal, to get the linear hexapeptide unit Boc-Gly-*l*-Pro-*l*-Tyr-*l*-Trp-*l*-Pro-Gly- OMe (**5**). The ester group of linear fragment was removed using LiOH and pentafluorophenyl (*pfp*) ester group was introduced. The Boc-group was removed using TFA and the deprotected linear fragment was now cyclized by keeping the whole contents at 0 °C for 7 days in the presence of catalytic amount of TEA/NMM/pyridine to get cyclic product (**6**) (Figure 1).

Synthesis of hexacyclopeptide was accomplished using solution-phase technique of peptide synthesis and structure of synthesized peptide was confirmed using spectral as well as elemental data. Disappearance of absorption bands at 1753, 1272 cm-1 and 1391, 1366 cm^-1 ^(C=O_str_ and C-O_str_, methyl ester group and C-H_bend_, *t*-butyl group) in FT-IR spectrum of **6 **clearly indicated cyclization of linear hexapeptide unit. This fact was further supported by disappearance of two singlets at *δ *1.53 and 3.62, corresponding to protons of *tert*-Butyl and methyl ester groups, in ^1^H NMR spectrum and disappearance of singlets at *δ *79.3, 28.9, and 50.9, corresponding to carbon atoms of *tert*-Butyl and methyl ester groups, in ^13^C NMR spectrum of **6**. Six signals between *δ *5.78-3.86 in the proton spectrum of **6 **suggested a peptide structure for the synthesized product, with these signals being attributable to the α - protons of all amino acid units. The ^1^H NMR spectrum of cyclized product showed presence of four broad singlets between *δ *9.85-7.65, 6.26 corresponding to the imino protons of the tyrosine, tryptophan, and two glycine moieties, remaining amino acids being two proline units, indicating similarity of the structure of the newly synthesized cyclohexapeptide with the natural molecule. Moreover, ^1^H/^13^C NMR spectra of the cyclized product **6 **showed characteristic peaks confirming the presence of all the 39 protons and 34 carbon atoms. Similar to natural molecule, presence of pseudomolecular ion peak at *m/z *658 corresponding to the molecular formula C_34_H_39_N_7_O_7_ in mass spectra of **6**, along with other fragment ion peaks resulting from cleavage at ‘Tyr-Pro’, ‘Pro-Trp’, ‘Gly-Gly’ and ‘Gly-Pro’ amide bond levels, showed exact sequence of attachment of all the six amino acid units in a chain (Figure 2). In addition, elemental analysis data of **6 **provided C, H, N% values (0.02) strictly in accordance to the molecular composition.

The synthesized cyclopeptide exhibited potent activity against pathogenic microbes *P. aeruginosa*, *K. pneumoniae*, *C. albicans, *and moderate level of activity against dermatophytes at 6 µg/mL, in comparison to reference drugs. However, neither compound **6 **nor its linear counterpart **5 **dispalyed any sort of bioactivity against *A. niger*. Moreover, compound **6 **displayed moderate to good level of antihelmintic activity against *M. konkanensis*, *P. corethruses *and *Eudrilus sp*. at 2 mg/mL, in comparison to standard drugs - mebendazole and piperazine citrate. In addition, the analysis of the pharmacological activity data revealed that hexacyclopeptide **6 **displayed a higher bioactivity against pathogenic microbes and earthworms than its linear form **5**, which is due to the fact that cyclization of peptides reduces the degree of freedom for each constituent within the ring and thus substantially leads to reduced flexibility, increased potency, and selectivity of cyclic peptides. Further, inherent flexibility of linear peptide **5 **can lead to different conformations which can bind to more than one receptor molecule, resulting in undesirable adverse effects.

## Conclusion

Diandrine C was synthesized in good yield using disconnection strategy. DIPC was found to be a good coupling agent for the synthesis of plant based hexacyclopeptide. The pentafluorophenyl ester was shown to be good ester group for the activation of the acid functionality of the linear hexapeptide unit. Using *N*-methylmorpholine as a base for cyclization of linear hexapeptide unit, maximum yield was obtained. Synthesized cyclopolypeptide showed remarkable activity against Gram-negative bacteria, *Candida *sp. and moderate to good level of antihelmintic activity against three species of earthworms. Gram-negative bacteria were found to be more sensitive towards the linear and cyclohexapeptides in comparison to Gram-positive bacterium. The newly synthesized cyclopeptide **6 **may prove to be a good candidate or a lead for future drugs with antimicrobial activity.
